# The Home of Rest for Horses

**Published:** 1889-11-09

**Authors:** 


### The Home of Rest for Horses.

Cabmen are not usually overburdened with idle sentimentality; and as they are witnesses to the practical benefits conferred on man and beast through this home, even a cautious man may believe "there is something in it." The home is situated at Sudbury, within two miles of Harrow, and is under the charge of a competent man, Mr. Sivell. The veterinary surgeon specially retained lives close by, and sees his "patients" twice a week, more frequently when necessary. On the occasion of our visit we inspected the large and airy loose boxes, also the warmer compartments for horses under special treatment, and "walked" the fifty acres of grass land, with the trees making a shelter here and there, where the animals are "turned out."

There were a good many of them, between thirty and forty, and the information given about them showed at once the purposes served by the home. It was a surprise to be told that a good-looking white horse belonged to a cabman, but if we had been there three weeks ago, when this animal came in, we were assured that the question of "good looks" would not have suggested itself. Then, there was a funny little dark pony, all mane and tail, blinking leisurely about, dividing his attention between the grass and the flies, and altogether suggesting that the home did business with Mr. Hengler, of the circus. We were assured that the children of a large family had all been taught riding by this pony, and that his mission in life being now quite over, he was sent here to end his days in peace. A spirited brown mare of fine "form" and intelligent appreciation of all that was passing also attracted attention. We were informed that her owner, now out of town, instead of leaving her in his stable, sent her out to the home "to be happy," and thoroughly recruit herself for fresh work. This accounts only for three out of the thirty animals or more, but it includes all the purposes, so far as we could ascertain, for which the home is used.

The practical and humanitarian aspects of this project are seen best in connection with what it secures for the horses of poor men—cabbies, costers, laundrymen and others. With these people the horse is a bread-winner, and is often, we fear, simply worked to death. This terrible cruelty is not always carelessly inflicted. It is simply that there is no help for it. The horse must work unceasingly, or his master and family must starve. Short-sighted policy, no doubt, as the animal breaks down long before his time, to the serious, sometimes irreparable, loss of the owner. The home takes the horse for a couple of weeks' rest at a small figure, and hires out an animal for work in the meantime, also on easy terms. The result has often been that poor men (and women) get their animals back improved and set up almost beyond recognition. When we come to the "old favourites" we enter upon debateable ground. People with horses no longer fit for work (and which the owners cannot decide to shoot) are glad to pay for them at the home. A great many people are to be reckoned under this head. There are many "old favourites" at the home, and always will be, we expect, although a member of the society (Col. Sir Francis Burdett, Bart.) says it would be far kinder to shoot animals when they are past work. The remaining purpose for which the home is used, securing pleasant surroundings for horses whilst their owners are out of town, points undoubtedly in the direction of kindness to the animals, but is, more than the other purposes, of the strictly commercial and business-like kind.

Miss Linds, of Westbourne Park, has been and is the inspiring spirit in this movement, and it is to her credit that kindness to over-worked horses, relief of their constant suffering, has been the principal motive in her action. We do not wonder at all that she has had sympathy and encouragement from the Society for the Prevention of Cruelty to Animals. What that society is often obliged to attempt through the expeditious method of the police-court should be still more certainly accomplished by Miss Linds's Home of Rest for Horses. The premises at Sudbury are admirable, as we have said, and not by any means over-taxed at present. If the movement is taken up on a larger scale, there is every means for developing the work at its present quarters.

